# FLO1K, global maps of mean, maximum and minimum annual streamflow at 1 km resolution from 1960 through 2015

**DOI:** 10.1038/sdata.2018.52

**Published:** 2018-03-27

**Authors:** Valerio Barbarossa, Mark A.J. Huijbregts, Arthur H.W. Beusen, Hylke E. Beck, Henry King, Aafke M. Schipper

**Affiliations:** 1 Radboud University, Institute for Water and Wetland Research, Department of Environmental Science, P.O. Box 9010, 6500 GL Nijmegen, The Netherlands; 2 PBL Netherlands Environmental Assessment Agency, Department of Nature and Rural Areas, P.O. Box 30314, 2500 GH The Hague, The Netherlands; 3 PBL Netherlands Environmental Assessment Agency, Department of Information, Data and Methodology, P.O. Box 30314, 2500 GH The Hague, The Netherlands; 4 Princeton University, Department of Civil and Environmental Engineering, Princeton, New Jersey, USA; 5 Unilever R&D, Safety and Environmental Assurance Centre, Colworth Science Park, Sharnbrook, Bedfordshire MK44 1LQ, UK

**Keywords:** Hydrology, Water resources, Freshwater ecology

## Abstract

Streamflow data is highly relevant for a variety of socio-economic as well as ecological analyses or applications, but a high-resolution global streamflow dataset is yet lacking. We created FLO1K, a consistent streamflow dataset at a resolution of 30 arc seconds (~1 km) and global coverage. FLO1K comprises mean, maximum and minimum annual flow for each year in the period 1960–2015, provided as spatially continuous gridded layers. We mapped streamflow by means of artificial neural networks (ANNs) regression. An ensemble of ANNs were fitted on monthly streamflow observations from 6600 monitoring stations worldwide, i.e., minimum and maximum annual flows represent the lowest and highest mean monthly flows for a given year. As covariates we used the upstream-catchment physiography (area, surface slope, elevation) and year-specific climatic variables (precipitation, temperature, potential evapotranspiration, aridity index and seasonality indices). Confronting the maps with independent data indicated good agreement (R^2^ values up to 91%). FLO1K delivers essential data for freshwater ecology and water resources analyses at a global scale and yet high spatial resolution.

## Background & Summary

Quantifying streamflow is critical to a variety of socio-economic and ecological analyses and applications^1–3^. Examples include the study of freshwater biodiversity patterns^4–7^, assessments of global water resources^8,9^, for example irrigation supply, hydropower or water footprinting^10–12^, analyses of the fate of pollutants^13^ and quantification of sediment fluxes^14,15^. Most of the stream reaches in the world are poorly or not monitored at all^16,17^, due to the inaccessibility of most headwaters and a lack of financial and human resources^18^, highlighted by a substantial decline in monitoring since the mid-1980s^17–19^. Streamflow is commonly quantified with process-driven global hydrological models (GHMs) and land surface models (LSMs)^20–24^. GHMs/LSMs are typically run at coarse spatial resolutions (~10 to 50 km), due to computational constraints, and consequently are unable to provide reasonable streamflow estimates for small rivers (defined here by Strahler stream order < 5), which comprise 94.6 % of the total stream length and riparian interface on the planet^25^. Streamflow data at higher spatial resolution would be highly beneficial for ecological applications and water resources assessment, for example understanding/modelling freshwater species distributions or modelling the fate and effects of pollutants in the aquatic environment^13,26–29^.

Compared to process-based models, data-driven models like regression equations and neural networks are more suited for generating high-resolution streamflow data with large spatial extent, thanks to their computational efficiency and relatively quick parameterization^30^. Data-driven models typically quantify streamflow based on upstream catchment characteristics related to topography, climate, land cover, and soils^30–33^. Data-driven approaches have been mostly employed at a local scale^34^. Recent studies demonstrated, however, the feasibility of applying a data-driven approach at a global scale, resulting in streamflow estimates that may have greater accuracy than the output of GHMs/LSMs^31,32^. Despite these encouraging results, consistent high-resolution global streamflow maps are not yet available.

Here we present FLO1K: a consistent dataset of global annual streamflow maps at 1 km resolution for each year in the period 1960-2015. Annual flow (AF) metrics include mean annual flow as well as minimum and maximum monthly flow for a given year. We produced the maps with feed-forward Artificial Neural Networks (ANNs) trained on yearly AF metric values from 6600 monitoring stations worldwide, using catchment-averaged covariates representing topography and climate. We delineated the upstream catchments based on the 1-km HydroSHEDS (www.hydrosheds.org) hydrography^35^, extended with Hydro1k (https://lta.cr.usgs.gov/HYDRO1K) for latitudes above 60°N not covered by HydroSHEDS, thereby achieving a global coverage (excluding Antarctica). For the training of the ANNs, we used 10 yearly values of mean, minimum and maximum AF per monitoring station and climate covariates for the corresponding years. We then constructed the AF metric maps by first computing for each year and each 30 arc seconds grid cell the upstream catchment-averaged covariates (which varied from year to year for climate), and then applying the trained ANNs. The streamflow is calculated for each terrain grid cell, i.e., it represents the potential in-channel discharge that would occur in the presence of a natural watercourse. The flow maps have a resolution 10 to 50 times higher than those typically produced using state-of-the-art GHMs/LSMs^36,37^ and global data-driven approaches^32^. For each of the three AF metrics, 56 yearly layers (1960-2015) are available packed in the NetCDF-4 format CF-compliant. In addition, we provide the FLO1K layers upscaled to 5 and 30 arc minutes resolutions for coarser-grain applications, including comparisons with GHMs/LSMs outputs. The FLO1K database can be downloaded from http://geoservice.pbl.nl/download/opendata/FLO1K and figshare (Data Citation 1).

## Methods

### General approach and streamflow network

**Figure 1:**
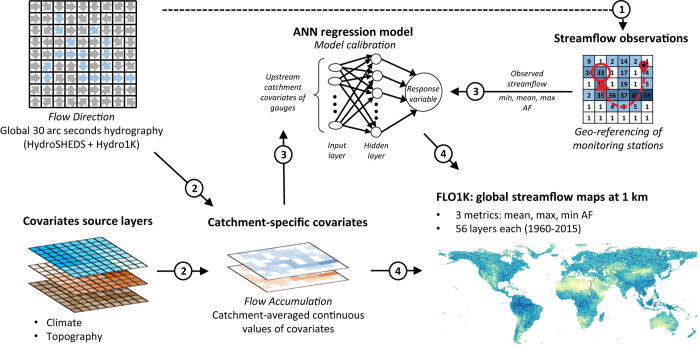
Schematic overview of the streamflow mapping procedure. The procedure consisted of four main steps: 1) monitoring stations (gauges) are geo-referenced based on the global hydrography, 2) catchment-specific covariates are compiled by aggregating climatic and physiographic variables over the upstream catchment of each cell, 3) ANNs are trained on monitoring data of AF metrics and covariates of the corresponding upstream catchment, 4) the trained ANNs are applied to the spatially-continuous covariates to create the global streamflow maps.

The procedure to generate the maps consisted of (i) model fitting, including observed streamflow data preparation, extraction of covariates, and training of the ANNs, and (ii) application of the ANNs to generate the global AF maps. Figure 1 provides a general outline of the procedure. We used the 30 arc seconds (~1 km) version of HydroSHEDS^35^ extended with Hydro1k for latitudes above 60°N to retrieve the drainage direction network and delineate the upstream catchment of each grid cell^38,39^. The HydroSHEDS hydrography is based on the National Aeronautics and Space Administration (NASA) Shuttle Radar Topography Mission (SRTM) digital elevation model (DEM)^40^, which covers the entire terrestrial land surface from latitudes 56°S to 60°N. To achieve a global spatial coverage, we extended HydroSHEDS with Hydro1k^38,39^, the latter being a United States Geological Survey (USGS) product derived from the GTOPO30 Digital Elevation Model (DEM) (https://lta.cr.usgs.gov/GTOPO30). The resulting drainage direction network is available at http://files.ntsg.umt.edu/data/DRT/.

### Streamflow observations

We derived mean, maximum and minimum AF values from flow records in the Global Runoff Data Centre (GRDC) database (www.bafg.de/GRDC)^41^. The GRDC comprises daily and monthly streamflow records from 9252 monitoring stations worldwide. The GRDC monitoring stations are not directly referenced on the hydrography employed in this study. This means that mismatched monitoring stations might encompass the wrong upstream catchment basin, which in turn may lead to errors when training the ANNs. As the GRDC dataset includes the estimated catchment area upstream of each monitoring station, we geo-referenced each station in order to match the most similar upstream area on the 30 arc seconds stream network, following the procedure previously used to allocate GRDC stations on the HydroSHEDS 15 arc seconds hydrography^42^. For each station, a new location is selected that minimizes discrepancies in catchment area and distance from the original location, within a 5 grid cells (~5 km) search radius. Out of the original 9252 monitoring stations, 285 were excluded as they did not report coordinates. Of the remaining 8967, 746 (~8%) were excluded because there was no matching catchment area within the search radius (based on a threshold of maximum 50% difference^42^). Out of the remaining 8221, 65% reported an area difference smaller than 5%, 15% had an area difference between 5% and 10%, and 20% had an area difference between 10% and 50%.

**Figure 2:**
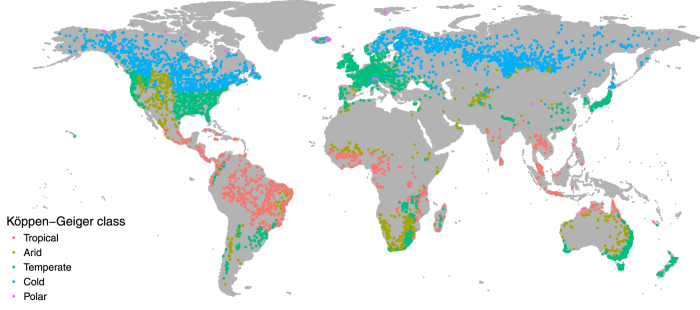
Distribution of the 6,600 GRDC stations monitored for at least 10 years in the period 1960-2015. Stations are coloured according to the Köppen-Geiger climate classification^67^.

We used the monthly records provided by the GRDC to calculate AF metrics for the period 1960-2015. We computed the mean AF for each year by averaging the 12 monthly values, and retrieved maximum and minimum AF by selecting the highest and lowest monthly values for each year, respectively. We considered only those years with a complete 12 months record and selected monitoring stations with at least 10 years of data from 1960 through 2015. The remaining set of stations totaled 6600 and were globally distributed as shown in Figure 2.

### Catchment-specific covariates

As covariates of the flow metrics we used topography and climate, which we retrieved from publicly available spatially explicit sources and then aggregated to the upstream catchment of each grid cell. The choice of the covariates set and source data was based on previous studies^30–34,43,44^, expert knowledge and data availability. A list of the covariates and related source databases is provided in Table 1.

**Figure 3:**
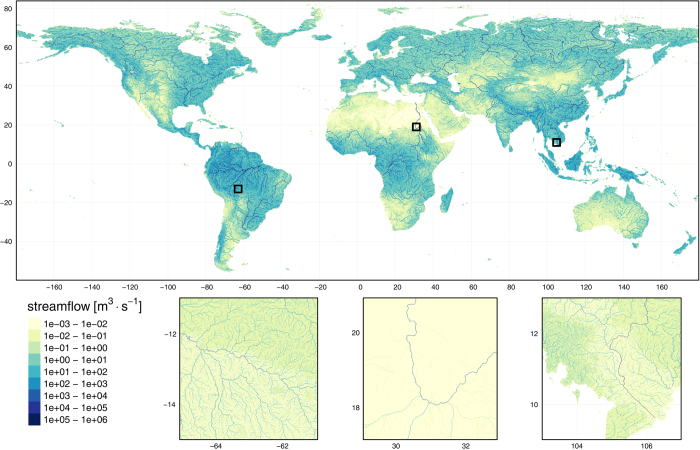
Long-term mean annual flow map overview. The long-term mean annual flow represents the average of the year-specific FLO1K maps for mean AF over the period 1960-2015. The global map has been upscaled using maximum-value resampling by a factor of 20 for clarity of visualization. Insets show the original 1 km resolution. Location of each inset is marked on the global map by a black square.

We calculated the area of the upstream catchment of each cell by summing the areas of the upstream grid cells. We derived the upstream catchment-averaged elevation from the SRTM DEM^40^ resampled at 30 arc seconds as provided by HydroSHEDS^35^, supplemented with the GTOPO30 DEM for areas lacking SRTM coverage, i.e., latitudes above 60°N. We transformed the elevation values by adding a constant value of 500 m to avoid negative values, the lowest being represented by the shores of the Dead Sea at 430 m below sea level. We employed the USGS slope map developed for the Prompt Assessment of Global Earthquakes for Response (PAGER) system^45^ to calculate upstream catchment-averaged surface slope values. This map is based on the same SRTM+GTOPO30 DEM and has been corrected for the discrepancy between ground units (arc degrees) and elevation units (meters)^45^.Figure 3

We derived the upstream catchment-averaged values for annual mean, maximum and minimum air temperature (T_air_) and precipitation (P), as well as potential evapotranspiration (PET), aridity index (AI) and seasonality index for P and PET, for every year over the period 1960-2015. For air temperature, we employed the Climate Research Unit (CRU) Time Series (TS) dataset^46^ (version 3.24.01; monthly temporal and 0.5° spatial resolution). For precipitation, we used the Multi-Source Weighted-Ensemble Precipitation (MSWEP) dataset^47^ (version 1.2; 3-hourly temporal and 0.25° spatial resolution; 1979-2015) supplemented with the Global Precipitation Climatology Centre (GPCC) Full Data Reanalysis^48^ (version 7; monthly temporal and 0.5° spatial resolution) prior to 1979. MSWEP merges a wide range of gauge, satellite, and reanalysis datasets to achieve precipitation estimates with greater accuracy than any other global dataset^47^. To combine the GPCC and MSWEP datasets, we rescaled the GPCC estimates such that the 1979-2013 mean of GPCC matched that of MSWEP. For each year and grid cell, we retrieved the mean annual value of T_air_ and P as the mean over the 12 monthly layers, and the minimum and maximum as the lowest and highest monthly values, respectively. We computed mean annual potential evapotranspiration from monthly T_air_ values following the temperature-based approach of Hargreaves et al.^49^ and employing the same CRU TS v. 3.24.01 source data for temperature. Similarly, we calculated seasonality index layers for P and PET as $X_{si}=X_{yr}^{-1}\sum^{\;}\left|X_{m}-X_{yr}/12\right|$, where *si*, *yr* and *m* stand for seasonality index, yearly and monthly values, respectively^50^. We downscaled the raster layers for the climate-related covariates to match the 30 arc seconds resolution of the hydrography using nearest-neighbour resampling. In addition, we calculated the aridity index for each year as PET/P, using mean annual P and PET.

To calculate the upstream catchment-average values of the covariates, we employed the TauDEM software (Terrain Analysis Using Digital Elevation Models, http://hydrology.usu.edu/taudem). TauDEM is an open-source C++ software explicitly designed to implement the flow algebra for large datasets, employing a Message Passing Interface (MPI, http://mpi-forum.org) to implement highly parallelized processing algorithms^51–53^. We extracted the covariates for the upstream catchment of each cell of the global hydrological network via the so-called flow accumulation technique (‘AreaD8’ in TauDEM). This technique considers each grid cell as a pour point and subsequently calculates the number of upstream grid cells or the sum of the attribute values of these upstream grid cells, using the flow direction map to delineate the watershed boundaries of the upstream catchment. To derive continuous upstream catchment-averaged values for the predictor variables, we divided the sum of the upstream covariate values by the total number of upstream grid cells at each pour point. To speed-up the calculations, we split the global flow direction layer into six continents (North America, South and Central America, Europe, Africa, Asia, Oceania). Adjacent continents (e.g., Europe and Asia) were separated along watershed boundaries.

### Training of Artificial Neural Networks

We quantified the relationships between the flow metrics and the covariates using artificial neural networks (ANNs), which have been widely used for hydrological modelling from local^54^ to global^32,33^ scales. We employed the feed-forward ANN algorithm based on the multi-layer perceptron structure with one hidden layer^55,56^ (Figure 1). We trained the ANNs based on year-specific values of mean, minimum and maximum AF, using the upstream-catchment topography and year-specific climate as covariates (Table 1). We applied a Box-Cox transformation to normalize the distributions of each variable (response and covariates)^57^. In addition, we standardized each distribution to zero mean and unit standard deviation, as required for the ANNs^56^. To avoid possible bias due to differences in monitoring intensity among the stations, we randomly picked 10 yearly values from those stations monitored at least 10 years across the 1960–2015 period. We then iterated the ANNs training 20 times, sampling different years from those stations having a record longer than 10 years. Prior to the training, we tuned the number of neurons of the hidden layer of the ANNs and the weights decay value to regularize the ANNs cost function, and therefore control for overfitting. To this end, we used 10-fold cross-validation (CV) whose folds were based on excluded monitoring stations, and identified the number of neurons and weights decay value that maximized the median coefficient of determination (R^2^) and minimized the median Root Mean Square Error (RMSE) of the testing set. As a result, we employed 20 neurons for the ANNs hidden layer and a weights decay value of 0.01.

### Generating mean, maximum and minimum AF global maps

We applied the ANNs model to produce 30 arc seconds maps with mean, maximum and minimum annual flow from 1960 through 2015 (Data Citation 1). For each grid cell, we computed the AF metrics as the median across the outputs of 20 trained ANNs and back-transformed the values to m^3^∙s^-1^.

We upscaled the 30 arc seconds layers to 5 and 30 arc minutes resolutions, in order to serve potential coarser-grain applications. We based the upscaled output on the 5 and 30 arc minutes flow direction grids produced by applying the dominant river tracing (DRT) algorithm to the same 30 arc seconds flow direction layer used in this study^38,39^. The 5 and 30 arc minutes flow direction grids are freely available for download at http://files.ntsg.umt.edu/data/DRT/. We upscaled the 30 arc seconds streamflow values by choosing the value of the cell that minimized the differences in upstream-drainage area between the native 30 arc seconds and the coarser resolution grid cell. For the 5 arc minutes grids it was necessary to employ a one-cell search radius to avoid losing connectivity.

### Code availability

The code used to generate the covariate data, geo-reference the monitoring stations, train the ANNs and generate the flow maps (Data Citation 1) was written and run in R version 3.3.2. TauDEM tools^52^ were used to produce the catchment-specific covariate layers and GDAL library^58^ functions were employed to handle the analyses on large raster data. The scripts are available on request.

The ensemble of trained ANNs are available as R objects (.rds) and as Portable Model Markup Language (PMML) objects for cross-platform compatibility (.pmml, http://dmg.org). The parameters used for the Box-Cox transformation and standardization of the variables employed by the ANNs are also available in CSV format.

## Data Records

The FLO1K dataset is a set of gridded layers packed as NetCDF-4 files freely available for download (Data Citation 1). For each of the three AF metrics, 56 yearly layers are available from 1960 through 2015, yielding a total of 168 layers. Each non-null cell represents the potential streamflow in m^3^∙s^-1^, stored as 32-bit floating point. Layers are in the WGS84 coordinate system with a cell size of 30 arc seconds (~ 1 km) and a global extent, including all continents except for Antarctica (90°N to 90°S latitude and 180°W to 180°E longitude). In addition, upscaled data are available at 5 and 30 arc minutes.

## Technical Validation

To evaluate the quality of the FLO1K maps, we run a 10-fold cross-validation for each of the 20 ANN runs, such that each observation was included in the test set once and by splitting the folds by stations. We assessed the overall map quality with R^2^ and RMSE calculated based on log-transformed values to evaluate the performance across the full spectrum of streamflow values (10^-3^-10^5^ m^3^∙s^-1^). Cross-validation results showed high agreement between training (90%) and independent testing (10%) data, with negligible variation among the replicates (Table 2).

**Figure 4:**
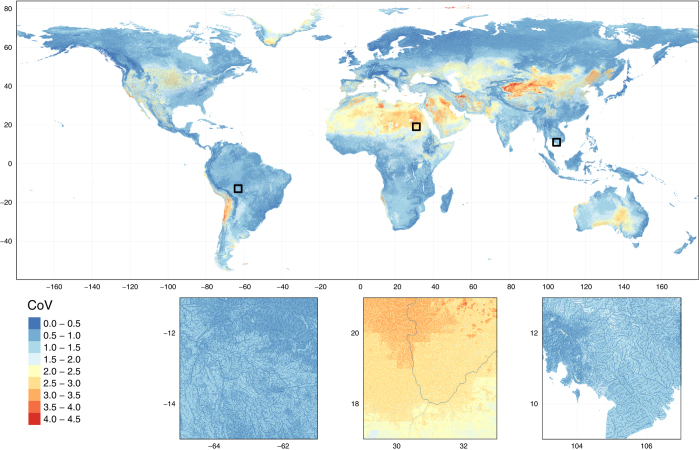
Uncertainty in mean AF due to differences in sub-sampling among the 20 ANN replicates. Uncertainty is expressed as coefficient of variation (CoV) averaged across the overall period 1960-2015. The global map has been upscaled using mode resampling by a factor of 5 for clarity of visualization. Insets show the original 1 km resolution. Location of each inset is marked on the global map by a black square.

We assessed the uncertainty per grid cell resulting from the sub-sampling of the monitoring stations, by computing the coefficient of variation (CoV) over the 20 replicates. Uncertainty was very low (CoV < 0.5) for the main river stems globally and smaller reaches in wet regions (Fig. 4). We found higher uncertainty (higher CoV values) for low streamflow values in dry areas, e.g., the upper basin of the Nile (central inset of Fig. 4). These higher CoV values likely reflect the lower number of streamflow observations available for calibrating the ANNs in these areas. The highest CoV values (> 3.5) were found in grid cells with a low number of upstream grid cells (typically <5) in dry areas. In these grid cells, most of the ANN replicates yielded zero-flow values whereas one or few replicates yielded close-to-zero values, resulting in a low mean yet large CoV across the 20 replicates.

We checked for potential bias in streamflow estimates in the northern hemisphere due to snowmelt delays, e.g., the contributing effect of snowfall in November-December of the previous year on the streamflow in May-June. To this end, we generated streamflow maps based on the US water year (November-October) for stations north of 40N and compared their performance to the original (calendar year-based) FLO1K maps. We tuned the ANNs ensemble and computed the streamflow fields adopting the US water year for both the streamflow data and the climate input variables. Differences in R^2^ between models based on calendar versus US water year were smaller than 0.01 and therefore considered negligible (Table 3).

## Usage notes

The FLO1K dataset reports the potential streamflow in m^3^·s^-1^ in each grid cell, i.e., the discharge that would occur if there were a natural watercourse. To avoid confusion, we emphasize that the estimates represent volumetric streamflow rather than specific runoff. As such, the estimates cannot directly be compared with outputs from climate or land surface models without a streamflow routing component.

We refrained from filtering the output to the actual stream network because there are multiple methods for stream network delineation^59–66^, which users of FLO1k may want to select or refine according to their needs. For global-scale analyses one might adopt an arbitrary upstream catchment area threshold in order to delineate the network (e.g., 25 upstream grid cells as in Hydrosheds^35^), as to our knowledge more refined methods have not yet been developed/tested.

The estimated maximum and minimum flow values for a given year reflect the highest and the lowest monthly values of that year. This does not give an indication about which months of the year belong to the maximum or minimum flow. The corresponding months might change from year to year based on the yearly distribution of the precipitation.

Users of the upscaled streamflow grids should keep in mind that these are contingent on the respective DRT flow direction layers^38,39^. Further, the accuracy of the upscaled grids has not been evaluated.

## Additional information

**How to cite this article**: Barbarossa, V *et al.* FLO1K, Global Maps of Mean, Maximum and Minimum Annual Streamflow at 1 km Resolution From 1960 Through 2015. *Sci. Data* 5:180052 doi: 10.1038/sdata.2018.52 (2018).

**Publisher**’**s note**: Springer Nature remains neutral with regard to jurisdictional claims in published maps and institutional affiliations.

## Supplementary Material



## Figures and Tables

**Table 1:** Comparison of R^2^ values for streamflow metrics based on calendar vs US water year.

Category	Variable description	Source	Annual metric	Unit	No. layers	Spatial resolution	Temporal coverage
Topography	Upstream catchment area	This study	-	km^2^	1	~ 1 km	-
	Elevation	SRTM^40^ + GTOPO30	-	m	1	~ 1 km	-
	Surface slope	USGS^45^	-	°	1	~ 1 km	-
Climate	Precipitation	MSWEP^47^ + GPCC^48^	Mean	mm·month^−1^	56×4	~ 25 km	1960–2015
			Minimum	mm·month^−1^			
			Maximum	mm·month^−1^			
			Seasonality index	-			
	Air temperature	CRU TS 3.24.01^46^	Mean	K	56×3	~ 50 km	1960–2015
			Minimum	K			
			Maximum	K			
	Potential evapotranspiration	This study	Mean	mm·month^−1^	56×2	~ 50 km	1960–2015
			Seasonality index	-			
	Aridity index	This study	-	-	56	~ 25 km	1960–2015
The comparison is based on 2,484 stations north of 40N latitude, monitored for at least 30 years in the period 1960-2015. The R^2^ was calculated from log-transformed values. LT: long term; YR: yearly.							

**Table 2:** Model performance statistics. The R^2^ and RMSE values represent medians (with standard deviations in brackets) of the 10-fold cross-validation of 200 replicates.

AF metric	R^2^		RMSE	
	Training	Testing	Training	Testing
Mean	0.92 (0.001)	0.91 (0.002)	0.32 (0.002)	0.34 (0.004)
Maximum	0.91 (0.001)	0.90 (0.002)	0.33 (0.002)	0.34 (0.005)
Minimum	0.85 (0.001)	0.83 (0.003)	0.48 (0.002)	0.51 (0.005)
Both R^2^ and RMSE were calculated from log-transformed values, therefore the RMSE is unitless.				

**Table 3:** Description of the predictor variables used as input for the modelling of AF.

	AF metric					
	Mean (LT)	Mean (YR)	Max (LT)	Max (YR)	Min (LT)	Min (YR)
Calendar year	0.975	0.953	0.969	0.936	0.933	0.885
US water year	0.975	0.955	0.969	0.942	0.928	0.879
The spatial resolution refers to the source data; for the analysis all variables were resampled to ~1 km.						
