# Erratum: FLO1K, global maps of mean, maximum and minimum annual streamflow at 1 km resolution from 1960 through 2015

**DOI:** 10.1038/sdata.2018.78

**Published:** 2018-04-24

**Authors:** Valerio Barbarossa, Mark A.J. Huijbregts, Arthur H.W. Beusen, Hylke E. Beck, Henry King, Aafke M. Schipper

*Scientific Data* 5:180052. doi:10.1038/sdata.2018.52 (2018); Published 27 March 2018; Updated 24 April 2018.

The titles and legends for Tables 1 and 3 were swapped in the original version of this publication. These have been corrected in both the HTML and PDF versions.

